# Editorial for the Special Issue on “Micro- and Nanofluidics for Bionanoparticle Analysis”

**DOI:** 10.3390/mi10090600

**Published:** 2019-09-12

**Authors:** Xuanhong Cheng, Yong Zeng

**Affiliations:** 1 Materials Science and Engineering/Bioengineering, Lehigh University, 5 E. Packer Ave, Bethlehem, PA 18015, USA; 2 Department of Chemistry, University of Kansas, 1251 Wescoe Hall Drive, Lawrence, KS 66045, USA

Bionanoparticles such as microorganisms and exosomes are recognized as important targets for clinical diagnostic and therapeutic applications as well as for food safety and environmental monitoring. Other nanoscale biological particles including liposomes, micelles, and functionalized polymeric particles are widely used in nanomedicine. The recent development of microfluidic and nanofluidic technologies has enabled lab-on-a-chip platforms for separating and analyzing bionanoparticles; however, many challenges exist before these platforms are widely adopted. For example, the complex composition of biological fluids creates a high background for detection. The small dimension and often low concentration of target species require significant amplification of the sensing signal. This special issue collects some recent discoveries and developments of micro- and nanofluidic strategies for the processing and analysis of biological nanoparticles. 

Eight papers are published in this special issue and cover the design of microfluidics for bioseparation [1,2,3,4,5,6], incorporation of sensors with microfluidics for particle detection [6,7], and modular devices with both functions [1,6,7]. Nanomaterials have been combined with microfluidics for both separation [5] and sensing [7] purposes. The special issue also includes a review on programmable paper-based microfluidics [8]. 

The collection of papers describe a wide spectrum of separation and sensing mechanisms. For example, Wang et al. [5] incorporated nanoporous membranes into microfluidic channels, and demonstrated the separation and enrichment of virus from biological fluids through nanofiltration. Yang et al. [6] took advantage of particle inertia in curved microfluidic channels to separate and contain particles in micro-tanks, which were further resolved by optical diffraction. Cheng et al. [7] coated a surface acoustic wave sensor with oxidized mesoporous carbon nanospheres to trap chemical compounds in smoke, where the coating increased the sensitivity and selectivity of the acoustic wave sensor. Boltz et al. [1] designed a split-flow device to perform continuous, in-line quality control of nanoparticle synthesis. Nanoparticles were detected by incorporating a fluorescence detector with the side channel. Soum et al. [4] fabricated conductive electrodes and dielectric films on a paper-based microfluidic chip to facilitate droplet manipulation based on electrowetting on dielectric materials. Luo et al. [3] studied the separation of non-magnetic particles in ferrofluids under a magnetic field. Liao et al. [2] employed optically induced dielectrophoresis to separate cancer cells from blood. The review paper by Soum [8] examined various paper-based microfluidics that are programmable and discussed the utility of such devices for the detection of biomarkers. 

We appreciate all of the authors who submitted papers to this special issue. We would also like to thank all of the reviewers for dedicating their time to help improve the quality of the submitted papers. The collection of papers will hopefully bring out more innovative ideas and fundamental insights to overcome the hurdles faced in the separation and analysis of bionanoparticles.
